# Interleukin Gene Expression in Mouse Preimplantation Development

**DOI:** 10.1155/1995/26830

**Published:** 1995

**Authors:** Nicole Gerwin, Gui-Quan Jia, Robert Kulbacki, José C. Gutierrez-Ramos

**Affiliations:** 1 The Center for Blood Research Inc. and the Department of Genetics Harvard Medical School Boston MA 02115 USA; 2 The Center for Blood Research Inc. 200 Longwood Avenue Boston MA 02115 USA

**Keywords:** Interleukins, cytokines, preimplantation development

## Abstract

Control of growth and differentiation during mammalian embryogenesis is regulated by growth factors from embryonic and/or maternal sources. Cytokines are polypeptide growth factors that are released by a variety of activated immune and nonimmune cells. To identify novel members of the cytokine family that could be involved in the growth and differentiation of the preimplantation embryo, we studied the expression pattern of several genes encoding cytokines and their receptors during mouse preimplantation development *in vitro* We found that poly(A)^+^ mRNAs for IL-1, IL-3, IL-6, IL-7, and TNF*α* are differentially expressed at several stages of mouse preimplantation development, including unfertilized oocytes. Immunostaining of preimplantation embryos using monoclonal antibodies specific for several cytokines and their receptors revealed that at least some of these mRNAs are translated into mature proteins during preimplantation development (IL-1, IL-6, and TNF*α*). Positive staining for IL-1 and IL-6 receptors was also detected at these stages of development. The controlled expression of these “inflammatory-type” cytokines and their receptors suggests a role for these growth factors during the early phases of mouse ontogeny.


*Developmental Immunology*, 1995, Vol 4, pp. 169–179
Reprints available directly from the publisher
Photocopying permitted by license only

© 1995 Harwood Academic Publishers GmbH
Printed in Singapore

### Interleukin Gene Expression in Mouse Preimplantation Development

NICOLE GERWIN,† GUI-QUAN JIA,† ROBERT KULBACKI,† and JOSÉ C. GUTIERREZ-RAMOS*

*The Center for Blood Research, Inc. and the Department of Genetics, Harvard Medical School, Boston, MA 02115, USA*

Control of growth and differentiation during mammalian embryogenesis is regulated by growth factors from embryonic and/or maternal sources. Cytokines are polypeptide growth factors that are released by a variety of activated immune and nonimmune cells. To identify novel members of the cytokine family that could be involved in the growth and differentiation of the preimplantation embryo, we studied the expression pattern of several genes encoding cytokines and their receptors during mouse preimplantation development *in vitro*. We found that poly(A)$^+$ mRNAs for IL-1, IL-3, IL-6, IL-7, and TNF$\alpha$ are differentially expressed at several stages of mouse preimplantation development, including unfertilized oocytes. Immunostaining of preimplantation embryos using monoclonal antibodies specific for several cytokines and their receptors revealed that at least some of these mRNAs are translated into mature proteins during preimplantation development (IL-1, IL-6, and TNF$\alpha$). Positive staining for IL-1 and IL-6 receptors was also detected at these stages of development. The controlled expression of these "inflammatory-type" cytokines and their receptors suggests a role for these growth factors during the early phases of mouse ontogeny.

KEYWORDS: Interleukins, cytokines, preimplantation development.

### INTRODUCTION

Preimplantation embryos grow and differentiate in the absence of exogenous factors *in vitro*, suggesting that endogenous factors are able of sustaining embryonic development during the first seven or eight cleavage divisions (Biggers, 1971). Direct evidence and indirect evidence indicate that embryos make growth factors. Indirect evidence comes, among others, from experiments in which cultured embryos from around the time of implantation were shown to produce transforming growth-factor like bioactivities that promoted anchorage-independent growth (Rizzino, 1985). Direct evidence comes from studies of gene expression, mainly performed at the blastocyst stage, that have documented the presence of transcripts for PDGF, TGF$\alpha$, TGF$\beta$, LIF, IL-6, and $\gamma$-IFN in preimplantation embryos (Rappolee et al., 1988; Murray et al., 1990; Rothstein et al., 1992).

Within the group of polypeptide growth factors, the more restricted cytokine family exhibits hormonelike properties that affect numerous organ systems (Kishimoto et al., 1994). They are expressed and act on both hematopoietic and nonhematopoietic cells. Mammalian cytokines include the interferons (IFNs), the interleukins (ILs), the colony stimulating factors (CSFs), the transforming growth factors (TGFs), as well as leukemia inhibitory factor (LIF), tumor necrosis factor (TNF), and lymphotoxin (LT). There is increasing evidence that cytokines participate in and modulate several events of early development and even pregnancy (Tartakovsky and Ben-Yair, 1991). At present, published data suggest the involvement of cytokines such as LIF during implantation (Stewart et al., 1992), and of TGF$\beta$ (Clark et al., 1988), and CSF-1 (Arceci et al., 1989; Chaouat et al., 1990) during postimplantation development. These polypeptide growth factors are believed to promote placental development, trophoblast proliferation, and, in the case of TGF$\beta$, to participate also in preventing harmful maternal antifetal immune responses. Much less is known about the role of cytokines in the preimplantation stages of development. LIF retards the differentiation of embryonic stem cells *in vitro*, but seems to

*Corresponding author: Present address: The Center for Blood Research, Inc. 200 Longwood Avenue, Boston, MA 02115.

promote the *in vitro* development of eight-cell embryos to the posthatching stages (Smith et al., 1988; Williams et al., 1988; Hilton, 1992). GM-CSF was demonstrated to have a beneficial effect on the *in vitro* development of implanting blastocysts from morulae (Tartakovsky and Ben-Yair, 1991). Another earlier study demonstrated that the *in vitro* growth of two-cell embryos was inhibited by a variety of cytokines (Hill et al., 1987).

Also, preimplantation embryos could be accessible targets for cytokines produced by the placental microenvironment. The interaction of preimplantation embryos with these biological-response modifiers or their induced products may well determine, modulate, or alter their programmed development. This notion may provide new and interesting insights into early events of mammalian development.

Our study focuses mainly on interleukins and is aimed to determine which genes encoding this subfamily of cytokines are expressed during preimplantation development. Also, it investigates if these interleukins are expressed at the protein level and correlates their expression with the presence of their specific receptors at different stages of preimplantation development.

### RESULTS AND DISCUSSION

Control of growth and differentiation during mammalian embryogenesis is regulated by growth factors from embryonic or maternal sources. Direct evidence for growth-factor transcripts of low copy number is difficult to obtain in preimplantation embryos, because thousands of embryos are required to detect high copy-number transcripts such as those coding for histones or actins by RNA blot analysis. Thus, for a detailed analysis of the cytokine gene-expression patterns in these stages, procedures were required that allow detection of both small amounts of mRNA as well as minor changes in interleukin (IL) gene expression. The polymerase chain reaction (PCR) was chosen because of its high sensitivity (Saiki et al., 1985). cDNA templates for PCR amplification were obtained using oligo(dT)-priming in the reverse-transcription (RT) reaction, in order to exclude immature or semidegraded poly-(A)less transcripts in the subsequent PCR analysis. Also, by demanding amplification between primers in different exons, the technique detects only properly spliced mRNAs. Although due to different affinities of the primers, no comparison can be made for the level of expression among different cytokines, reproducibility as well as parallel and simultaneous processing of all samples for a given pair of primers allows one to compare the pattern of expression for a given interleukin in different stages of development. To ensure further that differences in the quality of RNA preparations would not affect PCR amplifications of different samples differentially, titration of cycle number experiments was performed using β-actin oligos as primers and ethidium-bromide stained gels of PCR products obtained after 15, 20, 25, and 35 cycles of amplification (data not shown). Also, given the variability in the amount of RNA per cell in embryos at different stages of development (Piko and Clegg, 1982), we titrated the amount of RNA in each embryo preparation by amplifying the RNA of two ubiquitously expressed genes, β-actin and DHFR (data not shown). Thus, an equivalent amount of RNA (approximately equivalent to 75 ng of total RNA) was used finally for each PCR amplification. The authenticity of the PCR products detected was determined by hybridization to specific probes that lie internal to the primers used (Gutierrez-Ramos et al., 1992). The hybridization signals were proportional to the amount of product run on the gel, after amplification of a constant amount of cDNA (data not shown). Irrespective of the primers used, no PCR products were detected by hybridization if no RNA was included in the RT reaction prior to amplification (blank, Fig. 1). The detection of putative illegitimate transcription (Chelly et al., 1989) was avoided by using short amplifications (25 cycles), and could be ruled out by the reproducible lack of amplified bands in the negative control samples (data not shown). That equivalent amounts of total cDNA were available to the PCR primers was confirmed by the capacity to amplify equal amounts of (ubiquitously expressed) β-actin product from each sample (Fig. 1).

We first studied cytokine mRNA expression in mouse preimplantation embryos at different stages of development. Because oligo(dT) was used to prime the RT, the amplified fragments have to be derived from polyadenylated [poly(A)$^+$] RNA. We detected poly(A)$^+$ IL-1β, IL-3, and IL-7 transcripts in the fertilized oocyte, but no transcripts for IL-2, IL-4, IL-5, IL-6 and TNFα (Fig. 1; data not shown). Whereas no IL3 poly(A)$^+$ mRNA at all was detected in RNA preparations from two-cell stage embryos, detectable poly(A)$^+$ mRNAs for IL-1 and IL-7 were found at this stage of development. However, these

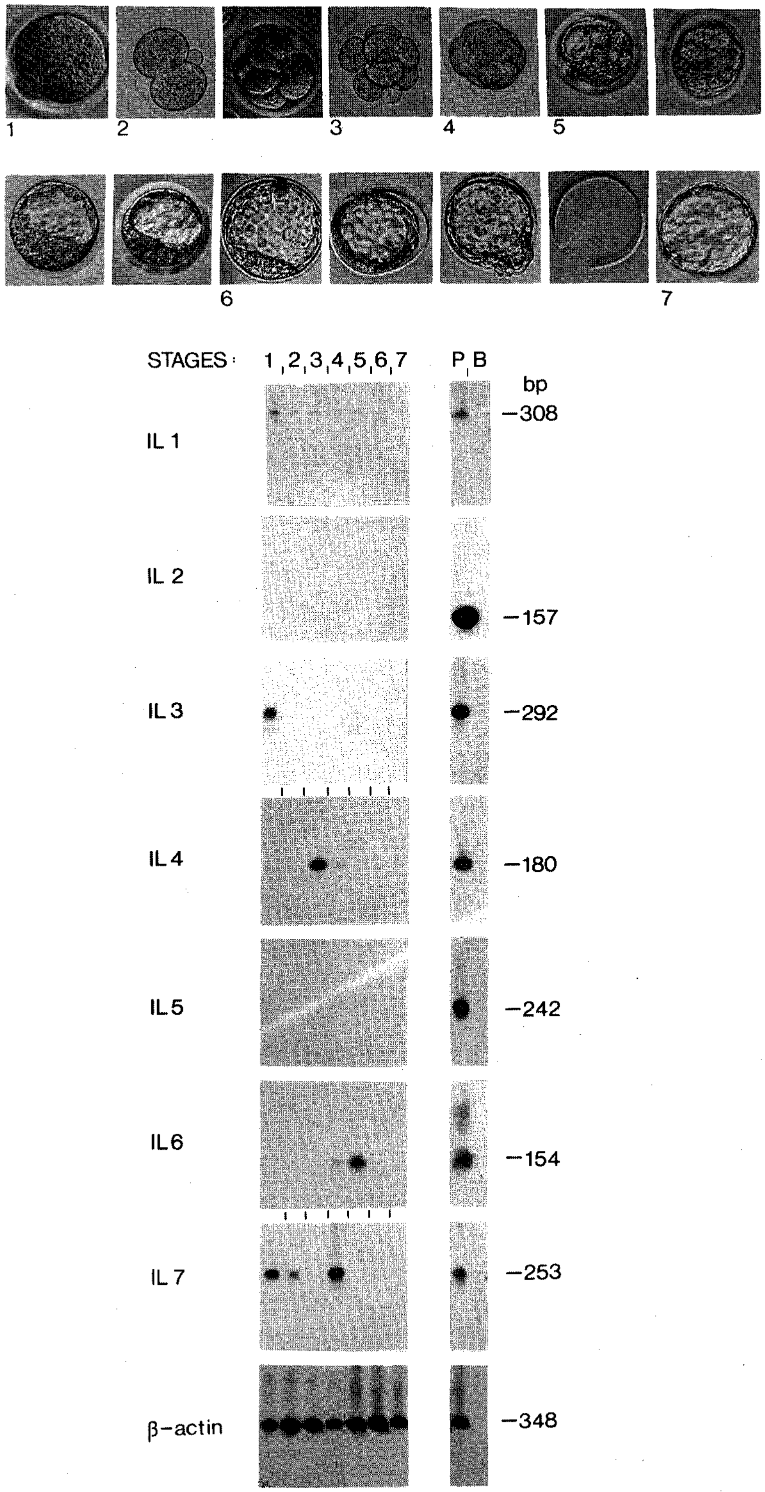

FIGURE 1. RT-PCR analysis of cytokine mRNA expression by mouse preimplantation embryos. The stage of the preimplantation embryos analyzed in each lane is indicated by a number, which corresponds to one picture in the top panel of the figure: 1, fertilized oocyte; 2, two-cell stage; 3, 16-cell stage; 4, compacted morula; 5, early blastocyst; 6, late blastocyst; and 7, expanded blastocyst. RT-PCR was performed as described in Materials and Methods. The size in base pairs of the amplified cDNA products is indicated in the margin. Two additional lanes for each cytokine designated as "P" and "B" show positive control and blank respectively.

mRNA species were reduced to approximately 10 and 20%, respectively, of the amount detected in the fertilized oocyte (Fig. 1).

The fact that most maternal mRNAs are cleaved and become undetectable around the two-cell stage (Flach et al., 1982) led us to explore the possible presence of mRNA for IL-1, IL-3 and IL-7 in unfertilized oocytes. Some maternal mRNAs are present in the unfertilized oocyte in a poly(A)less form (Proudfoot, 1991; Simon et al., 1992). To ensure that both poly(A)$^+$ and poly(A)less transcripts could be detected in unfertilized oocytes during these new series of PCR reactions for interleukin detection, we followed two different strategies to generate cDNA templates. cDNAs from unfertilized mouse embryos were generated using oligo(dT) or random hexamers as primers for RT. Amplification of oligo(dT)-primed cDNAs with specific cytokine primers only detects poly(A)$^+$ mRNA, whereas the use of random hexamers or 3′-specific primers (data not shown) ensures that both polyadenylated and non-polyadenylated mRNAs are detected. Figure 2 shows a series of experiments aimed to detect specific transcripts for IL-1, IL-3, and IL-7 in the poly(A)$^+$ and/or poly(A)less RNA pools from fertilized and unfertilized oocytes. We found that mRNAs for these genes were detectable in unfertilized oocytes only when random-hexamer-primed cDNAs were used for the PCR and not when oligo(dT)-primed material was used. This indicates that mRNA for these three cytokines is present in the unfertilized oocyte in a poly(A)less form. In contrast, the mRNAs for these three genes are present in a poly(A)$^+$ form in the fertilized oocyte as indicated by the detection of these mRNA species independently of the priming strategy used [(oligo(dT)-primed or random hexamer-primed)] for the reverse transcription of mRNA from fertilized oocytes. These findings together with the disappearance/reduction of IL-1, IL-3, and IL-7 mRNA around the two-cell stage suggest that these transcripts are of maternal origin. Very early events in embryonic development take place in the absence of transcription and depend on information provided by maternal mRNA and protein (Dworkin and Dworkin-Rastl, 1990). In *Drosophila*, development is under maternal control for the first 13 nuclear divisions, after which the embryonic genome is transcriptionally activated. Embryonic transcription in *Xenopus* is not required until the midblastula transition when the embryo consists of approximately 4000 cells. In mammalian development, the

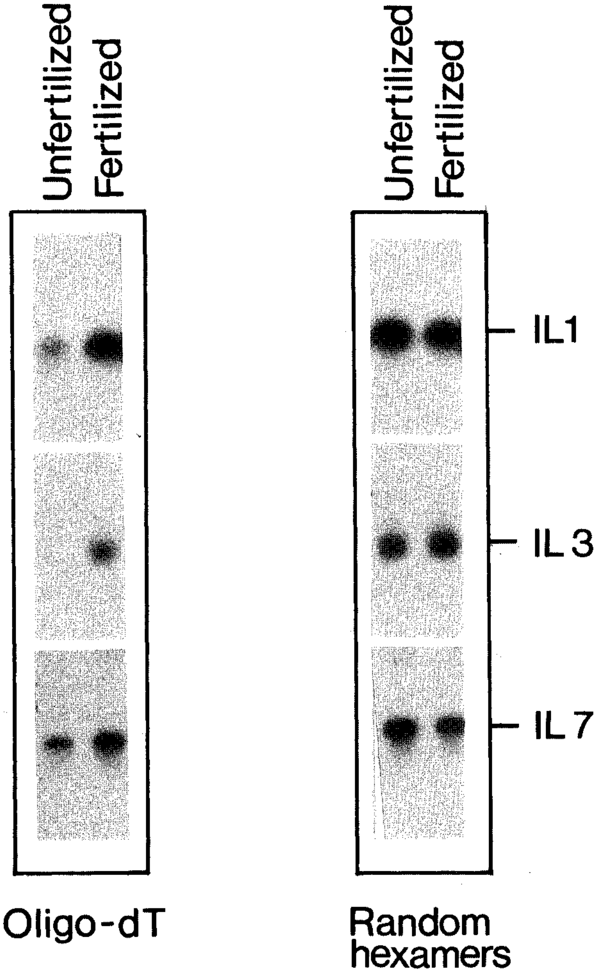

FIGURE 2. RT-PCR analysis of cytokine mRNA expression by unfertilized and fertilized oocytes. Fertilized and unfertilized oocytes were analyzed for the presence of specific mRNA for the indicated interleukins. For these experiments, RNA was reverse transcribed, priming either with oligo(dT) or with random hexamers as indicated in the figure.

period of exclusive maternal control is shorter, and in the mouse is believed to last for only one cell division (Johnson, 1981). Little if any transcription is detectable until the two-cell stage, and $\alpha$-amanitine, a potent inhibitor of RNA-polymerase II, fails to block the first cell division. Completion of the first cell division is thought to require translation of at least some maternally inherited mRNAs. These experiments have suggested that the maternal inheritance is required to traverse the first cycle and to activate transcription from the genome of two-cell mouse embryos. The finding of IL-1 and IL-3 transcripts in the unfertilized oocyte stage deserves special attention due to the features of these two growth factors. IL-1 is a pleiotropic differentiation factor capable of inducing the expression of other genes such as c-*fos*, c-*abl*, and other protooncogenes (Lipsky et al., 1983; Botazzi et al., 1990; Callard and Gearing, 1994). It has been detected in the trophoblast and placental tissue of murine embryos as well as in later stage human embryos (Crainie, 1990). IL-3 is known to have an impact on the survival and differentiation of early hematopoietic progenitors and specific effects on more committed cell lineages such as mast cells (Arai et al., 1990; Callard and Gearing, 1994).

We found that cytokine mRNA expression was also regulated at later stages of preimplantation development. As stated before, IL-7 mRNA was detected in unfertilized and fertilized oocytes, disappeared around the two-cell stage, and reappeared in compacted morulas (Fig. 1). According to our previous experience with these PCR primers (Gutierrez-Ramos et al., 1992), the observed differences in the amount of IL-7 mRNA between oocytes and morulas indicate that IL-7 transcripts are tenfold more abundant in the morula than in the fertilized oocyte. IL-7 is known to induce the differentiation of immature lymphocytes (Henney, 1989; Callard and Gearing, 1994; Komschlies et al., 1994), and to activate directly growth regulatory genes such as N-*myc* or c-*myc* (Morrow et al., 1992).

Transcripts for IL-6 were identified from the eight-cell stage of development on (Fig. 1). The detection of IL-6 transcripts is interesting because IL-6 is a multifunctional cytokine. It plays a major role in the inflammatory responses and is the primary inducer of acute phase proteins (Kishimoto, 1989; Callard and Gearing, 1994). This cytokine promotes the differentiation and/or proliferation *in vitro* and *in vivo* of cells belonging to several lineages at different stages of their maturation (Kishimoto, 1989). In addition, IL-6 is known to have effects on the expression of several other genes, including IL-1 (Shabo et al., 1988). Our results confirm previous reports describing the presence of IL-6 transcripts at the blastocyst stage (Murray et al., 1990; Rothstein et al., 1992), and extends them by defining the profile of expression of IL-6 at different stages of preimplantation development.

Although zygotic genome transcription is very active at the blastocyst stage (Shultz, 1986), it seems unlikely that preimplantation embryos express these cytokine genes promiscuously. In fact, four other cytokine genes (IL-2, IL-3, IL-4, and IL-5), which are expressed in several tissues in adult mice and/or in other ontogenic stages, were not found to be expressed during preimplantation development in our experiments.

Rappolee et al., (1990) had classified the accumulation patterns of growth factors that do not belong to the interleukin family into three classes. In one class, maternal transcripts apparently dissappeared and were resynthesized in the zygote. In the second class, some transcripts were not present maternally, but appeared as the result of zygotic transcription. In the third class, transcripts such as for the metalloproteinase stromyelsin apparently survived the breakdown of maternal RNAs that occurs in the two-cell stage (Rappolee et al., 1990). The mRNAs for cytokines that have been analyzed in this work seem to belong to the two first classes, unless the slow disappearance of IL-7 around the two-cell stage is interpreted as survival of this RNA species.

Because the transcription of growth-factor mRNAs is not invariably coupled with the translation into proteins (Assoian et al., 1987), it was necessary to determine whether the transcripts that were detected by RT-PCR were translated into mature proteins in the preimplantation embryo. However, these studies were somehow restricted by the availability of specific monoclonal antibodies only for some cytokines (see Material and Methods). Immunocytochemistry experiments revealed the presence of IL-1, IL-6, and TNF$\alpha$ proteins in mouse preimplantation embryos. Preimplantation embryos ranging from the one-cell stage to the blastocyst stage were stained with the different antibodies indicated in the Material and Methods section. Fertilized oocytes (Fig. 3, panel I) did not show any positive staining with antibodies specific for IL-4 (Fig. 3c) or IL-6 (Fig. 3d), whereas staining with antibodies specific for IL-1 resulted in a strong positive signal (Fig. 3a), which was not seen in its isotype-matched irrelevant negative control antibody (Fig. 3b). The same phenotype was also observed during the two-cell stage (Fig. 3, panel II). This interesting pattern of IL-1 staining, together with the availability of specific antibodies against the receptors for IL-1 prompted us to determine if receptors for IL-1 (IL-1R) could be present at the same developmental stages as its ligand. In fact, positive staining for the IL-1 receptors (type I and/or type II) was observed both in oocytes and two-cell stage embryos (Figs. 3e and 3k). No staining was observed at these stages of preimplantation development when specific antibodies for IL-6 receptors were used (Figs. 3f and 3l). IL-1 has a different intracellular transport pathway would eventually participate in the endoderm lineage formation.

The detection of IL-1 and IL-6 proteins in mouse preimplantation embryos prompted us to determine the possible presence of TNF$\alpha$, which together with IL-1 and IL-6 forms the group of inflammatory cytokines (Beutler and Cerami, 1989; Callard and Gearing, 1994). TNF$\alpha$ protein was not detected before the late blastocyst stage. Staining of late and expanded blastocysts (panel IV) with anti-TNF$\alpha$ antibodies resulted in diffuse positive staining of all blastomeres (data not shown). Specific mRNA for TNF was also detected by RT-PCR at these late preimplantation stages (data not shown). The pattern of spatial expression of IL-1 (Fig. 3r) and IL-1R (Fig. 3u) at the expanded blastocyst stage deserves special attention. Whereas low levels of IL-1 were detected uniformly in all blastomeres at this stage, its receptor(s) were brightly expressed only in few blastomeres, resulting in a much more restricted pattern of staining (Fig. 3u). Staining of late and expanded blastocysts with antibodies specific for IL-6 or with the irrelevant antibody mentioned previously did not result in positive signal (Figs. 3t and 3s, respectively). However, staining of late and expanded blastocysts with specific antibodies for IL-6R, resulted in diffused positive staining in virtually all blastomeres (Fig. 3v). Soon after blastocoel formation and expansion of the blastocyst, the embryo hatches and implants in the uterine wall. During implantation, extensive differentiation, proliferation, and tissue remodeling take place, parts of which resemble inflammatory reactions. TNF$\alpha$ and IL-1 transcripts have been identified in different organs of normal rats, and among many other cell types TNF-$\alpha$ is likely to be produced by cells in the uterus and placenta during pregnancy. Northern blotting experiments have identified TNF-$\alpha$ transcripts in murine placental RNA (Crainie, 1990). In addition, biologically active TNF$\alpha$ and IL-1 have been reported in human decidua and conditioned media from human embryos contain immunoreactive TNF-$\alpha$ (Witkin et al., 1991).

Most data on growth-factor action in nonmamalian early embryos implicate their function in differentiation rather than in mitosis (Rappolee et al., 1990). Other growth factors, like bFGF or TGF$\beta$ appear to be morphogens responsible for the induction of mesoderm at the blastulation stage, as has been shown in *Xenopus* (Kimelman and Kirschner, 1987; Weeks and Melton, 1987). However, the early development of the mouse has several properties that distinguish it from that of the frog. For example, the mouse egg is small, has little yolk, and quickly activates zygotic transcription after fertilization. The functions of inflammatory cytokines in preimplantation embryos are as yet unknown, but might include differential effects on the growth of blastocyst-derived cells and their expression of classI MHC, stimulation of angiogenesis, and implantation of the hatched blastocyst. In addition, the implantation process itself is characterized by very specific changes in the receptive endometrium, which include local increase in capillary permeability and release of histamines and prostaglandins (Psychoyos, 1986; Yelavarthi et al., 1991). These changes reflect an inflammatorylike process and as such, the proinflammatory cytokines IL-1, IL-6, and TNF$\alpha$ could play an important role. One possibility is that these cytokines bind to receptor cells in the oviduct and/or uterine endometrium and activate, for example, epithelial cells or macrophages to secrete substances that promote development and subsequent implantation of the early embryo. The survival of mice that have been made deficient for these genes (Kopf et al., 1994; Pfeffer et al., 1993; and Jia and Gutierrez-Ramos, in preparation) suggests that singly these cytokines are not essential for embryonic development. However, it remains to be determined if the concerted action of two or more of these factors is required for the proper development of the mouse preimplantation embryo.

### MATERIALS AND METHODS

### Preparation of Mouse Embryos and Cultures

Isolation and culture of mouse embryos were performed as described (Hogan et al., 1986). Three-to-six-week-old (C57BL/6xDBA/2)F1 females kept on a light cycle from 5 A.M. to 7 P.M., were superovulated by intraperitoneal injection of 5 U per mouse of gonadotropins from pregnant mare's serum at 2 P.M., followed 48 hr later by injection of 5 U per mouse of human chorionic gonadotropin. These mice were mated overnight with (C57BL/6xDBA/2)F1 males, and embryos were harvested from plugged females between 7 A.M. and 9 A.M. the following morning. Embryos were devoided of surrounding cumulus mass cells by digestion with hyaluronidase, which was followed by five consecutive washes in M2 medium (Hogan et al., 1986). Embryos were cultured in 90 μl drops of M16

medium (Hogan et al., 1986), covered by paraffin oil and kept in 7.5% $CO_2$ at 37°C. Differential interference contrast microscopy and microphotography were performed with a Nikon Diaphot microscope. Alternatively, embryos at the two-cell and eight-cell stages (second and third day of pregnancy, respectively) were isolated by flushing M2 medium, through oviducts. Blastocysts on the fourth day of pregnancy were isolated by flushing the uterine horns.

### Immunofluorescence

Preimplantation embryos were fixed immediately after collection or after a 3-to-6-day culture period. The zona pellucida was not removed. Fixation was performed in 2% paraformaldehyde/0.1% glutaraldehyde in 0.1% phosphate buffered saline (PBS), pH 7.4, for 30 min at room temperature (RT). After washing, fixed embryos were treated for 5 min at RT with a 0.05% solution of sodium borohydride in PBS without $Ca^{++}$ and $Mg^{++}$ and washed again. Cells were permeabilized by incubation for 10 min at RT in 0.1% Triton X-100 in PBS. After washing, when the specimens were not previously cultured, the embryos were preincubated for 60 min at RT in a 0.5% w/v solution of BSA in DMEM (Hepes buffered, 25 mM). This solution was also used to dilute the first antibody and the second fluorescein isothiocyanate (FITC)-coupled goat anti-rat IgG (H + L) antibody 1:60 (Jackson Immunoresearch, Bar Harbor, ME). The incubation time for each antibody was 1 hr at RT. Four washes of 10 min were performed between each incubation. Finally, the specimens were embedded in a 90/10 mixture of moviol and PBS containing paraphenylenediamine (Johnson and Nogueira-Araujo, 1981).

Specimens were viewed in a Bio-Rad Lasersharp MRC-600 Confocal Light Scanning Microscope (CLSM), and hard copies were obtained in a Sony color video printer. Microscope settings were kept identical when comparing test and control samples.

### Antibodies

The following monoclonal antibodies were used: anti-IL-2 (hybridoma S4B6); anti-IL-4 (hybridoma 11B11; Ohara and Paul, 1985); anti-IL-6 (Genzyme, Boston, MA); anti-IL-6R (which is specific for the α-chain of the receptor; Genzyme, Boston); anti-TNFα (Genzyme); anti-IL-1 (British Biotechnology, Oxford UK); anti-IL-1R, which recognizes IL-1R type I and II (Genzyme, Boston). As irrelevant antibodies isotype-matched negative controls were used [anti-Il-2Rα; hybridomas PC61 (ratIgG) or 7D4 (ratIgM]). As a second step reagent, a 1:60 dilution of a goat anti-ratIgG FITC-labeled antibody (Jackson Immunoresearch) was used.

### RT-PCR Analysis

Preimplantation embryos were washed in PBS twice, resuspended in 40 to 100 µl of DEPC-water containing 40 U of RNAsin (Promega, Madison, WI) and boiled for 10 min. The crude material obtained was RQ1-DNAse-treated (New England Biolabs, Boston) for 30 min at 37°C. After inactivation of the DNAse, the crude material was reverse transcribed using oligo$(dT)_{12-15}$ as a primer and MMLV reverse transcriptase in a 20 µl reaction (Land et al., 1983). Four microliters of the reverse-transcribed material (equivalent to approximately four embryos) were used directly for the first determination of amplification reactions, which were performed using specific primers for ubiquitous genes to titrate the amount of RNA in each sample (see Results). Conditions for PCR were as follows: In a 50 µl reaction, 25 nmol of each primer (see what follows), 25 µM of each dGTP, dATP, dTTP and dCTP; 50 mM KCl, 10 mM Tris-HCl, pH 8.3; 1.5 mM $MgCl_2$; 1 mg/ml gelatin; and 1 U Taq DNA polymerase ("AmpliTaq" Perkin-Elmer/Cetus, Norwalk, CT). Primers used were the following: IL-1α (nucleotides 1573–1595 and 1881–1861; Lomedico et al., 1984), IL-2 (nucleotides 203–217 and 370–346; Yokota et al., 1985), IL-3 (nucleotides 286–310 and 578–555; Yokota et al., 1984), IL-4 (nucleotides 231–255 and 411–387; (Lee et al., 1986), IL-5 (nucleotides 1182–1206 and 1424–1400; Yokota et al., 1987); IL-6 (nucleotides 581–605 and 735–711; Chiu et al., 1988), IL-7 (nucleotides 1146-1170 and 1446–1422; (Tokunaga et al., 1986) and β-actin (nucleotides 886–910 and 1234–1210; (Noma et al., 1986). To avoid unspliced mRNA or DNA (DNAse-resistant) amplification, each primer of every pair is located in different exons. In addition, the absence of contaminating DNA was confirmed by performing PCR amplifications of intronic sequences of the α-actin gene, as previously described (Van Meerwijk et al., 1990) Reactions were incubated in Perkin-Elmer/Cetus DNA thermal cycler for 25 cycles (denaturation 30 s, 94°C; annealing 30 s, 55°C; extension 1 min, 72°C) following the manufacturer's recommendations. Seven microliters of the PCR reaction were loaded on a 1.5% agarose gel. Specific amplification was ensured by the size of the product, as shown on the gel relative to known standards and by probing the Southern blots with the following $^{32}$P-labeled cDNA probes: IL-1$\alpha$ (0.9 kb BamHI-BamHI; Lomedico et al., 1984); IL-2 (1 kb XhoI-XhoI; Yokota et al., 1985); IL-3 (375 bp HindIII-XbaI; Yokota et al., 1984); IL-4 (300 bp RsaI-RsaI; (Van Snick et al., 1988), IL-5 (430 bp XhoI-XhoI; Yokota et al., 1987); IL-6 (650 bp EcoRI-BglII); IL-7 (450 bp SstI-HindIII; Samaridis et al., 1991); and β-actin (1.1 kb PstI-PstI). The probes were isolated fragments labeled with 32P-dATP by using a random-primer DNA labeling kit (Boehringer Mannheim, Indianapolis, IN). In every case hybridization was performed under the same conditions using the same amount of cpm ($3 \times 10^6$ cpm/ml of hybridization buffer) followed by standard washings. In every case, 45 min RT exposures are shown. Longer exposure did not change the shown pattern of expression. Longer exposure of the IL-2- or IL-5-probed filter did not show any additional bands. Titrations of cycle number showed that the amount of specific amplified products increased approximately logarithmically up to between 35 and 40 cycles. Thus, to keep reactions within nonlimiting conditions and yet obtain detectable signals, 25-cycle amplifications were chosen. PCR products obtained with β-actin primers after 15, 20, 25, and 35 cycles of amplification were detected with ethidium-bromide stained gels (data not shown). Densitometric analysis of these β-actin amplifications showed that the samples had a coefficient of variance of 0.19, which was insignificant compared to the changes in message levels observed in different stages of development. As an additional control, 45 cycles were performed in each experiment (not shown) and processed in parallel to the "25 cycles experiment", which is shown in Fig. 1. This set of parallel experiments showed the reproducibility of the assay and as well confirmed the nonsaturating conditions used for the "25-cycle experiments" shown here. The sensitivity of our PCR conditions was estimated by titrating a known number of copies of the genes analyzed here (corresponding plasmids) containing the relevant primer sites with cDNA known to be negative for the message of interest. These experiments revealed that by performing 25-cycle amplifications followed by hybridization with 45-min exposure, we could reproducibly detect between 10 and 1000 cDNA molecules depending of the primers used (Gutierrez-Ramos et al., 1992). Due to different efficiencies of the primers, comparisons cannot be made among different ILs in terms of levels of expression. However, because all samples for a given pair of primers were processed simultaneously, in parallel with master mixes (including every reaction component but templates), loaded on the same gel, and hybridized on the same filters under non-saturating conditions, comparisons can be made for the expression of a given IL in different stages of development.

Two separate controls were performed in every experiment: A blank tube (every component but RNA template) and one positive control (1 μg of RNA from PMA + Ionomycin-stimulated spleen cells for IL-2, IL-3, IL-4, IL-5 expression, from LPS-stimulated P388D1 macrophage cell line for IL-1 and IL-6 expression, from the J558L myeloma cell line transfected with the mouse cDNA encoding IL-7 (Samaridis et al., 1991) for IL-7 expression.

### ACKNOWLEDGMENTS

The authors are indebted to Mrs. Carina Olsson for excellent technical assistance and to the Basel Institute for Immunology for fostering the early phases of this project. The Basel Institute for Immunology was founded and is supported by Hoffmann-LaRoche, Ltd. Basel, Switzerland. This work has been funded in part with 1PO1HL 148675-02. N.G. is a recipient of a DFG postdoctoral fellowship. G.-Q.J. is a recipient of a WHO-PABO training grant. J.C. G.-R. is the Amy C. Potter fellow.

### REFERENCES

Arai K., Lee F., Miyajima A., Miyatake S. Arai N., and Yokota T. (1990). Cytokines: Coordinate regulation of immune and inflammatory responses. Annu. Rev. Biochem. **59**: 783–836.

Arceci R.J., Shanahan F., Stanley E.R., and Pollard J.W. (1989). Temporal expression and location of colony-stimulating factor 1 (CSF-1) and its receptor in the female reproductive tract are consistent with CSF-1 regulated during placental development. Proc. Natl. Acad. Sci. USA **86**: 8818–8822.

Assoian R.K., Fleurdelys B.E., Stevenson H.C., Miller P.J., Madtes D.K., Raines E.W., Ross R., and Sporn M.B. (1987). Expression and secretion of type β transforming growth factor by activated human macrophages. Proc. Natl. Acad. Sci. USA **84**: 6020–6024.

Beutler B., and Cerami A. (1989). The biology of cachectin/TNF-α primary mediator of the host response. Annu. Rev. Immunol. **7**: 625–655.

Biggers J.D. (1971). The biology of the blastocyst (Chicago: University of Chicago Press), pp. 319–327.

Bottazzi B., Nobili N., and Mantovani A. (1990). Expression of *c-fos* protooncogene in tumor-associated macrophages. J. Immunol. **144**: 4878–4882.

Callard R., and Gearing A. (1994). The cytokine facts book (London: Academic Press), pp. 36–73.
Chaouat G., Menu E., Clark D.A., Minkowski M., and Wegmann T.G. (1990). Control of fetal survival in CBAxDBA/2 mice by lymphokine therapy. J. Reprod. Fertil. **89**: 447–458.
Chelly J., Concordet J.P., Kaplan J.C., and Kahn A. (1989). Illegitimate transcription: transcription of any gene in any cell type. Proc. Natl. Acad. Sci. USA **86**: 2617–2621.
Chiu C.-P., Moulds C., Coffman R.L., Rennick D., and Lee F. (1988). Multiple biological activities are expressed by a mouse interleukin 6 cDNA clone isolated from bone marrow stromal cells. Proc. Natl. Acad. Sci. USA **85**: 7099–7103.
Clark D.A., Falbo M., Rowley R.B., Banwatt D., and Stedronska-Clark J. (1988). Active suppression of host vs graft reaction in pregnant mice. J. Immunol. **141**: 3833–3840.
Crainie, E. (1990). Inflammatory cytokines in the placental environment and in late human embryos. Biol. Reprod. Immunol. **19**: 85.
Dworkin M.B., and Dworkin-Rastl E. (1990). Functions of maternal mRNA in early development. Mol. Reprod. Dev. **26**: 261–267.
Flach G., Johnson M.H., Braude P.R., Taylor R.A.S., and Bolton V.N. (1982). The transition from maternal to embryonic control in the 2-cell mouse embryo. EMBO J. **1**: 681–686.
Gardner R.L. (1985). Regeneration of endoderm from primitive ectoderm in the mouse embryo: Fact or artifact? J. Embryol. Exp. Morphol. **88**: 303–326.
Gutierrez-Ramos J.C., Olsson C., and Palacios R. (1992). Interleukin (IL1 to IL7) gene expression in fetal liver and bone marrow stromal clones: Cytokine-mediated positive and negative regulation. Exp. Hematol. **20**: 986–990.
Henney C.S. (1989). Interleukin 7: Effects on early events in lymphopoiesis. Immunol. Today **10**: 170–173.
Hill J.A., Haimovici F., and Anderson D.J. (1987). Products of activated lymphocytes and macrophages inhibit mouse embryo development *in vitro*. J. Immunol. **139**: 2250–2254.
Hilton D.J. (1992). LIF: Lots of interesting functions. Trends Biochem Sci. **17**: 72–76.
Hogan B., Constantini F., and Lacy H. (1986). Manipulating the mouse embryo (Cold Spring Harbor, NY: Cold Spring Harbor Laboratory Press) pp. 89-150.
Johnson G.D., and Nogueira-Araujo G.M. (1981). A simple method of reducing the fading of immunofluorescence during microscopy. J. Immunol. Methods **43**: 349–350.
Johnson M.H. (1981). The molecular and cellular basis of preimplantation mouse development. Biol. Rev. Camb. Philos. Soc. **56**: 463–498.
Karasuyama H. and Melchers F. (1988). Establishment of mouse cell lines which constitutively secrete large quantities of interleukin 2, 3, 4 or 5, using modified cDNA expression vectors. Eur. J. Immunol. **18**: 97–104.
Kimelman D., and Kirschner M. (1987). Synergistic induction of mesoderm by FGF and TGF-β and the identification of an mRNA coding for FGF in the early Xenopus embryo. Cell **51**: 869–877.
Kishimoto, T. (1989). The biology of interleukin-6. Blood **74**: 1–10.
Kishimoto T., Taga, T., and Akira S. (1994). Cytokine signal transduction. Cell **76**: 253–262.
Komschlies K.L., Gregorio T.A., Gruys M.E., Back T.C., Faltyneck C.R., and Wiltrout R.H. (1994). Administration of human IL-7 to mice alters the composition of B-cell lineage cells and T-cell subsets, enhances T-cell function, and induces regression of metastases. J. Immunol. **152**: 5776–5784.
Kopf M., Baumann H., Freer G., Freudenberg M., Lamers M., Kishimoto T., Zinkernagel R., Bluethmann H., and Koehler G. (1994). Impaired immune and acute-phase responses in interleukin-6-deficient mice. Nature **368**: 339–342.
Land H., Grez M., Hauser H., Lindenmaier, W. and Schutz G. (1983). Synthesis of ds-cDNA involving addition of dCMP tails to allow cloning of 5′-terminal mRNA sequences. Meth. Enzymol. **100**: 285–292.
Lee F., Yokota T., Otsuka T., Meyerson P., Villaret D., Coffman R., Mosmann T., Rennick D., Roehm N., Smith C., Zlotnik A., and Arai K.-I. (1986). Isolation and characterization of a mouse IL cDNA clone that expresses B-cell stimulatory factor 1 activities and T-cell and mast-cell stimulatory activities. Proc. Natl. Acad. Sci. USA **83**: 2061–2065.
Lipsky P.E., Thompsom P.A., Rosenwaser L.J., and Dinarello C.A. (1983). The role of interleukin 1 in human B-cell activation: inhibition of B-cell proliferation and the generation of immunoglobulin-secreting cells by an antibody against human leukocytic pyrogen. J. Immunol. **130**: 2708–2714.
Lomedico P.T., Gubler U., Hellman C.P., Dukovich M., Giri J.G., Pan Y.C., Collier K., Semionow R., and Mizel., S.B. (1984). Cloning and expression of murine interleukin-1 cDNA in Escherichia coli. Nature **312**: 458–462.
Morrow M.A., Lee G., Gillis S., Yancopoulos G.D., and Alt F.W. (1992). Interleukin-7 induces N-*myc* and c-*myc* gene expression in normal precursor B lymphocytes. Genes Dev. **6**: 61–70.
Murray R., Lee F., and Chiu C.P. (1990). The genes for leukemia inhibitory factor and interleukin-6 are expressed in mouse blastocysts prior to the onset of hemopoiesis. Mol. Cell. Biol. **10**: 4953–4956.
Noma Y., Sideras P., Naito T., Bergsted-Lindquist S., Azuma C., Severinson E., Tanabe T., Kinashi T., Matsuda F., Yaoita Y., and Honjo T. (1986). Cloning of a cDNA encoding the murine IgG1 induction factor by a novel strategy using SP6 promoter. Nature **319**: 640–646.
Ohara J., and Paul W.E. (1985). Production of a monoclonal antibody to and molecular characterization of B-cell stimulatory factor-1. Nature **315**: 333–336.
Pfeffer K., Matsuyama T., Kundig T.M., Wakeham A., Kishihara K., Shahinian A., Wiegmann K., Ohashi P.S., Kronke M., and Mak T.W. (1993). Mice deficient for the 55 kd tumor necrosis factor receptor are resistant to endotoxic shock, yet succumb to L. monocytogenes infection. Cell **73**: 457–467.
Piko L., and Clegg K.B. (1982). Quantitative changes in total RNA, total poly(A) and ribosomes in early mouse embryos. Dev. Biol. **89**: 362–378.
Proudfoot N. (1991). Poly(A) signals. Cell **64**: 671–674.
Psychoyos A. (1986). Uterine receptivity for nidation. Annu. N.Y. Acad. Sci. **476**: 36–42.
Rappolee D., Sturm K., Schultz G., Pedersen R., and Werb Z. (1990). The expression of growth factor ligands and receptors in preimplantation mouse embryos. In: Early embryo development and paracrine relationships (New York: Alan Liss), pp.11–25.
Rappolee D.A., Brenner C.A., Schultz R., Mark D., and Werb Z. (1988). Developmental expression of PDGF, TGF-α, and TGF-β genes in preimplantation mouse embryos. Science **241**: 1823–1825.
Rizzino A. (1985). Early mouse embryos produce and release factors with transforming growth factor activity. In Vitro Cell Dev. Biol. **21**: 531–537.
Rothstein J.L., Johnson D., DeLoia J.A., Skowronski J., Solter D., and Knowles B. (1992). Gene expression during preimplantation mouse development. Genes Dev. **6**: 1190–1201.
Rubartelli A., Cozzolino F., Talio M., and Sitia R. (1990). A novel secretory pathway for interleukin-1β, a protein lacking a signal sequence. EMBO J. **9**: 1503–1510.
Saiki R.K., Scharf S., Faloona F., Mullis K.B., Horn G.T., Erlich H.A., and Arnheim N. (1985). Enzymatic amplification of β-globin genomic sequences and restriction site analysis for diagnosis of sickle cell anemia. Science **230**: 1350–1354.
Samaridis J.G., Casorati G., Gutierrez-Ramos J.C., Iglesias A., Trauneker A., and Palacios R. (1991). Development of lymphocytes in interleukin-7-transgenic mice. Eur. J. Immunol. **21**: 453–457.

Sander B., Andersson J., and Andersson U. (1991). Assessment of cytokines by immunofluorescence and the paraformaldehyde-saponin procedure. Immunol. Rev. **119**: 65–93.

Shabo Y., Lotem J., Rubinstein M., Revel M., Clark S.C., Wolf S.F., Kamen R., and Sachs L. (1988). The myeloid blood cell differentiation-inducing protein MGI-2A is interleukin-6. Blood **72**: 2070–2073.

Shultz G.A. (1986). Experimental approaches to mammalian embryonic development, Rossant J., and Pedersen, R.A. Ed. (New York: Cambridge University Press), pp. 239–265.

Simon R., Tassan J.P., and Richter J.D. (1992). Translational control by poly(A) elongation during Xenopus development: Differential repression and enhancement by a novel cytoplasmic polyadenylation element. Genes Dev. **6**: 2580–2591.

Smith A.G., Heath J.K., Donaldson D.D., Wong G.G., Moreau J., Stahl M., and Rogers D. (1988). Inhibition of pluripotential embryonic stem cell differentiation by purified polypeptides. Nature **336**: 688–690.

Stewart C.L., Kaspâr P., Brunet L.J., Bhatt H., Gadi I., Kontgen F., and Abbondanzzo S.J. (1992). Blastocyst implantation depends on maternal expression of LIF. Nature **359**: 76–79.

Tartakovsky B., and Ben-Yair E. (1991). Cytokines modulate preimplantation development and pregnancy. Dev. Biol. **146**: 345–352.

Tokunaga K., Taniguchi H., Yoda K., Shimizu M., and Sakiyama S. (1986). Nucleotide sequence of a full-length cDNA for mouse cytoskeletal β-actin mRNA. Nucl. Acid Res. **14**: 2829.

Van Meerwijk J.P, Bluethmann H. and Steinmetz M. (1990). T-cell specific rearrangement of T-cell receptor beta transgenes in mice. EMBO J. **9**: 1057–1062.

Van Snick J., Cayphas S., Szikora J.P., Renauld J.C., Van Roost E., Boon T. and Simpson R.J. (1988). cDNA cloning of murine interleukin-HP1: Homology with human interleukin 6. Eur. J. Immunol. **18**: 193–197.

Weeks D.L., and Melton D.A. (1987). A maternal mRNA localized to the vegetal hemisphere in Xenopus eggs codes for a growth factor related to TGF-β. Cell **51**: 861–867.

Williams R.L., Hilton D.J., Pease S., Willson T.A., Stewart C.L., Gearing D.P., Wagner E.F., Metcalf D., Nicola N.A. and Gough N.M. (1988). Myeloid leukaemia inhibitory factor maintains the developmental potential of embryonic stem cells. Nature **336**: 684–687.

Witkin S.S., Liu H.C., Davis O.K., and Rosenwaks Z. (1991). Tumor necrosis factor is present in maternal sera and embryo culture fluids during *in vitro* fertilization. J. Reprod. Immunol. **19**: 85–93.

Yelavarthi K.K., Chen H.L., Yang Y.P., Cowley Jr. B.D., Fishback J.L., and Hunt J.S. (1991). Tumor necrosis factor-α mRNA and protein in rat uterine and placental cells. J. Immunol. **146**: 3840–3848.

Yokota T., Arai N., Lee F., Rennick D., Mosmann T., and Arai K. (1985). Use of a cDNA expression vector for isolation of mouse interleukin 2 cDNA clones: expression of T-cell growth-factor activity after transfection of monkey cells. Proc. Natl. Acad. Sci. USA **82**: 68–72.

Yokota T., Coffman R.L., Hagiwara H., Rennick D.M., Takebe Y., Yokota K., Gemmell L., Shrader B., Yang G., Meyerson P. et al. (1987). Isolation and characterization of lymphokine cDNA clones encoding mouse and human IgA-enhancing factor and eosinophil colony-stimulating factor activities: relationship to interleukin 5. Proc. Natl. Acad. Sci. USA **84**: 7388–7392.

Yokota T., Lee F., Rennick D., Hall C., Arai N., Mosmann T., Nabel G., Cantor H., and Arai K. (1984). Isolation and characterization of a mouse cDNA clone that expresses mast-cell growth factor activity in monkey cells. Proc. Natl. Acad. Sci. USA **81**: 1070–1074.

